# The COVID-19 Resource Centre: an invaluable tool for primary care

**DOI:** 10.29173/jchla29742

**Published:** 2024-08-01

**Authors:** Anne Dabrowski, Taylor Moore, Tupper Bean, Lena Salach, Katie Hagel, Lindsay Bevan, Pippy Scott-Meuser, Amanda van Hal, Christina De Longhi, Kelly Lang-Robertson, Ellen Tulchinsky

**Affiliations:** 1 Director, Information Services, The Centre for Effective Practice, Toronto, ON; 2 Coordinator, Information Services, The Centre for Effective Practice, Toronto, ON; 3 Chief Executive Officer, Santero Group; 4 Chief Executive Officer, The Centre for Effective Practice, Toronto, ON; 5 Vice President, Programs, The Centre for Effective Practice, Toronto, ON; 6 Director, Clinical Appropriateness, The Centre for Effective Practice, Toronto, ON; 7 Director, Digital, The Centre for Effective Practice, Toronto, ON; 8 Manager, Project Solutions, Q2 Solutions; 9 Specialist, Information Services, The Centre for Effective Practice, Toronto, ON; 10 Information Research Manager, The Centre for Effective Practice, Toronto, ON; 11 Manager, Information Services, The Centre for Effective Practice, Toronto, ON

## Abstract

**Background:**

In response to the COVID-19 pandemic, the Ontario-based Centre for Effective Practice (CEP) established the COVID-19 Resource Centre (CRC) in March 2020. This platform rapidly became a critical source of clinical and practice guidance for primary care providers, highlighting the importance of effective information synthesis during public health emergencies.

**Description:**

The article discusses the development of the CRC, emphasizing the application of librarianship principles in navigating the challenges posed by the pandemic's information overload and the scarcity of evidence. It outlines the strategies for literature searching, appraisal, and evidence synthesis that were employed to ensure the content's accuracy and utility. The CRC's evolution is presented within the context of its goal to efficiently bridge the gap between evidence and clinical practice, underscoring the collaborative efforts and innovative methodologies that contributed to its success.

**Outcomes:**

The CRC has served as an invaluable resource, attracting close to 185,000 visitors from Ontario, across Canada, and internationally. According to survey feedback, 89% of users reported enhanced knowledge of COVID-19 evidence and policies, and 87% stated that the vaccine information directly informed their practice. These statistics underscore the CRC's role in supporting informed decision-making among healthcare providers.

**Discussion:**

The CRC marked the CEP's first foray into real-time evidence-based tool development. Facing challenges of expanding information volumes, an unpredictable information landscape, and the need for swift adaptation to new developments, the CRC emerged as a critical resource, enhancing credibility for the CEP, and fostering new partnerships. This journey underscores the importance of librarianship skills—critical appraisal, evidence synthesis, and knowledge translation—in enhancing service delivery.

## Introduction

With the onset of the COVID-19 pandemic, primary care clinicians in Ontario were bombarded by, at times, conflicting, inefficient, impracticable, or misleading information accumulating at a staggering rate. Without streamlined information specific to primary care that could keep pace with the evolving landscape, clinicians could not distinguish how to best support themselves, their practices, their patients, or the broader healthcare system.

For 20 years, the Centre for Effective Practice (CEP) has been one of the leading independent healthcare behaviour-change partners in Canada and a trusted source of high-quality, evidence-based clinical supports for primary care providers. Through clinician education tools and resources, point-of-care clinical decision supports, one-on-one practice facilitation, and a suite of other services, the CEP drives meaningful change that improves health system outcomes and advances health system priorities. To support the CEP’s mission to close the gap between evidence and practice, the organization employs a team of full-time, in-house medical librarians, whose day-to-day work includes not only methodology, searching, appraisal, and curation, but critical analysis, synthesis, and knowledge translation.

In March of 2020, the CEP quickly responded to meet the crisis of our target audience. With dual expertise in evidence and knowledge translation, the CEP’s librarians led the vision and design of streamlined, easy-to-read, hyper-current and practical guidance for Ontario primary care providers on clinical, operational, and administrative information related to COVID-19. With only three weeks’ planning time, the CEP launched the COVID-19 Resource Centre (CRC) in mid-April 2020.

The CEP was not unique in the system in recognizing the essential need to fill information gaps. The COVID-19 Evidence Synthesis Network and the COVID-19 Science Table launched within the first few months of the pandemic. Trusted government and professional organizations rapidly produced guidance. Health librarians, policymakers and clinicians relied on Public Health Ontario’s excellent “What we know so far about…” series.

Deliberate commitment to speed and improvisation is what differentiated the CRC from other clinical knowledge translation initiatives. With a one-to-three-day content development cycle and a creative, need-based approach, CRC quickly became one of the most-used resources for primary care in the province.

The success of the CEP’s approach and the demonstrable value it provided to our audience translated to new partners, processes and product streams for the CEP. This article's purpose is to detail select features of the CRC approach that were successful and can be applied to support any kind of public health information need. It will highlight aspects of the approach and methodology that can be adapted and scaled in any setting, and present knowledge translation and evidence synthesis as high-value skill sets for any health librarian. Finally, it will illustrate how high-quality knowledge translation products are powerful exhibits of the intrinsic value of health librarianship.

The Description section will articulate at a high level the service and its audience, including platform and products created, governance and staffing structure, and methodology. The Outcomes section will provide quantitative and qualitative data describing overall project outcomes. The Discussion section will share the legacy of the CRC for the CEP, weaknesses of the CRC and things to do differently in the future, and the lessons learned.

## Description

**Fig. 1:**
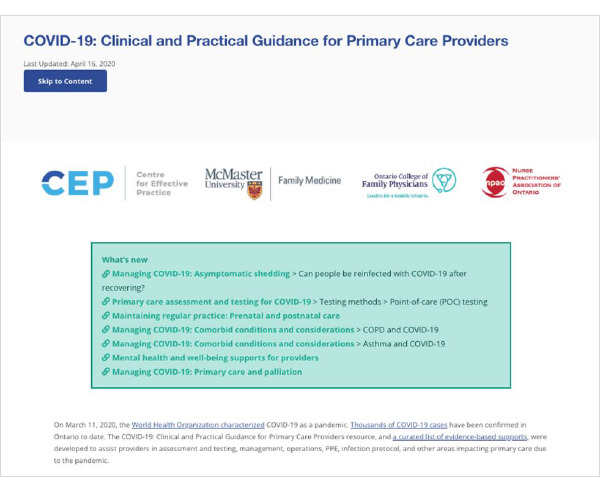
CRC landing page circa April 17, 2020

**Fig. 2:**
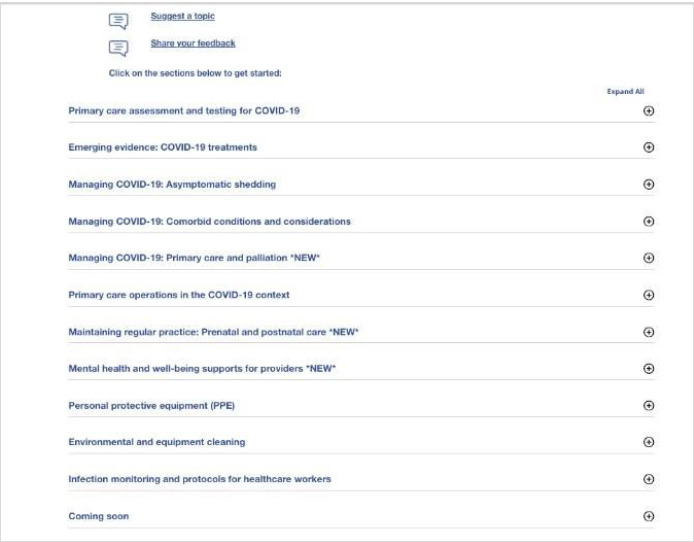
CRC topics circa April 2020

In operation from April 2020 to April 2024, the CRC was an HTML-based suite of clinical and practice guidance about COVID-19, funded by the Ontario Ministry of Health (MOH) under the CEP’s Knowledge Translation in Primary Care initiative and targeting Ontario primary care family physicians and nurse practitioners. In response to significant gaps identified by the CEP and MOH, the CRC launched as a single central resource page with 13 sub-sections (Figures 1-2):

- Primary Care Assessment and Testing For COVID-19
- Emerging Evidence – COVID-19 Treatments
- Managing COVID-19: Asymptomatic Shedding, Comorbid Conditions and Considerations, Primary Care and Palliation
- Primary Care Operations in the COVID-19 Context
- Maintaining Regular Practice: Prenatal And Postnatal Care
- Mental Health and Wellbeing Supports for Providers
- Personal Protective Equipment
- Environmental And Equipment Cleaning; Infection Monitoring and Protocols for Healthcare Workers
- A “Coming soon” list of additional new sections in development

**Fig. 3:**
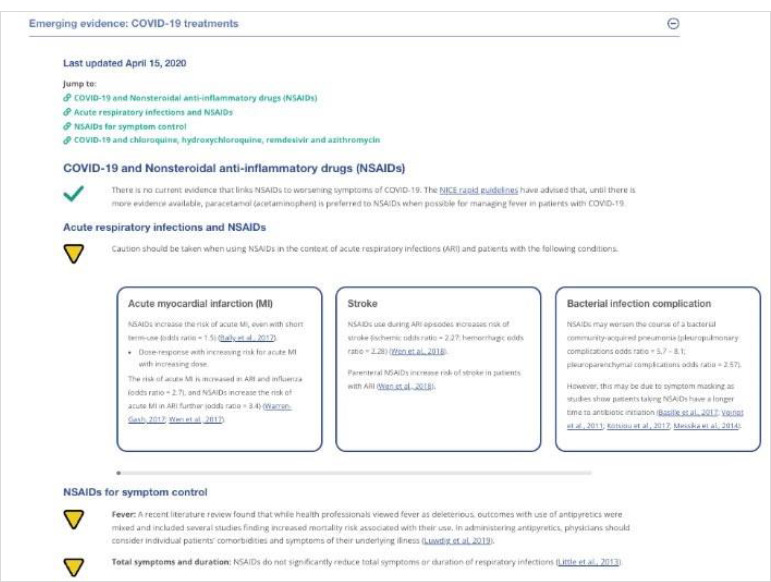
Example CRC April 2020

**Fig. 4:**
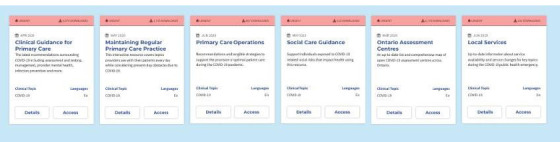
Expanded CRC June 2020

**Fig. 5:**
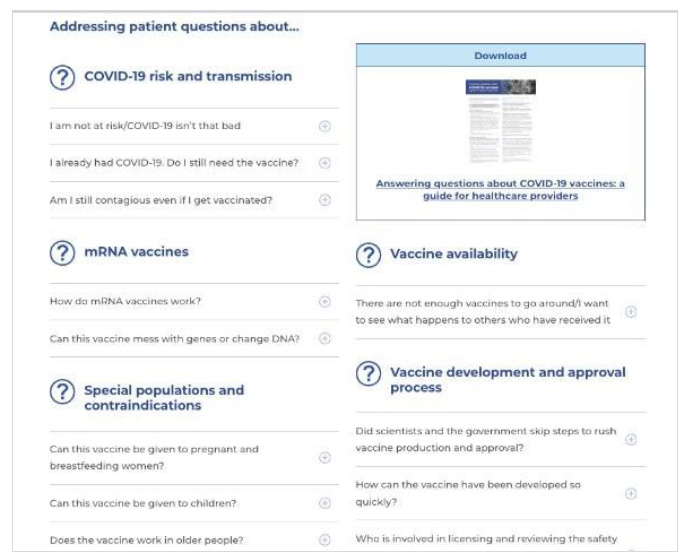
Product examples - Vaccine Q&A

**Fig. 6:**
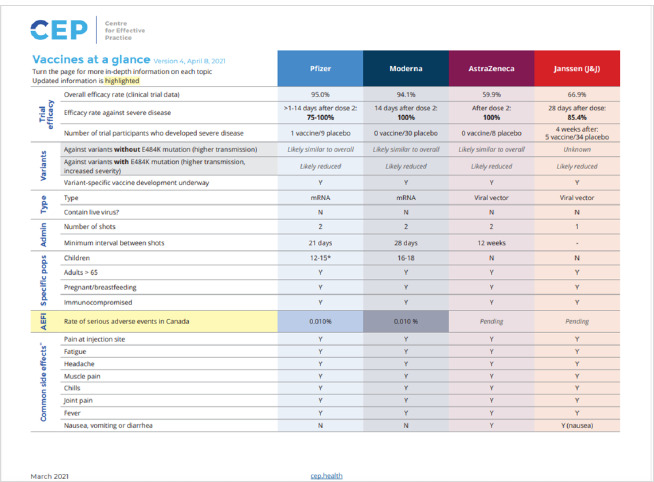
Product examples - two-page evidence summary PDF

Most CRC content was written directly into the HTML platform, to enable easy editing and updating for staff and straightforward searching and navigation for users. However, a variety of different products were developed depending on the audience's needs for a particular topic. Products offered by the CRC between 2020-2024 included but were not limited to: health system navigation supports; clinical workflows; short, rapid evidence syntheses; public health and provincial government guidance syntheses; contextualizing and fact-checking media reports, rumors and misinformation; operational guidance and checklists; one and two-page graphically designed PDFs; Q&As for clinicians and patients (Figures 3-6). While primarily written in English, specific key resources were also provided in French.

### Roles throughout CRC development and operation

Collaboration & partnerships: CEP leadership including past and present CEOs, VPs, the Director of Clinical Appropriateness, and the Manager of the Knowledge Translation in Primary Care initiative developed and refined comprehensive governance and feedback structures, identifying and engaging diverse expert and health system perspectives. In addition, significant efforts were directed towards forging new partnerships and collaboration opportunities. Continuous engagement with expert partners was instrumental in creating the CRC’s highly actionable, focused, and trustworthy guidance.

Evidence & content: The Director of Information Services, in collaboration with CEP internal leadership, led evidence synthesis and content development for the CRC’s knowledge products. She was responsible for products, scope, methodology, process, project management, staffing, and delivery. Staff medical librarians reviewed all content for accuracy, practicality, and adherence to methodology before acceptance for inclusion on the website. Staff at the CEP, including clinical design experts and primary care pharmacists (Academic Detailers), were the primary researchers and writers for the CRC. With the support of the Director and staff librarians, 2-person teams worked together to track, draft and update content on each topic and subtopic. For more details on the delivery process, see the Methods section.

Digital platform: CEP's Director Digital created the HTML platform for the CRC, managing its overall design as well as daily operational needs, such as hundreds of weekly updates. The CRC's rapid expansion through new tools and partnerships, necessitated a creative, innovative, and adaptable approach to maintain ease of navigation and usability.

### External partners and collaborators

Numerous individuals, institutions, organizations, and associations lent their time and expertise to the development of the CRC. They helped leadership to identify needs and suggest topics; provided expert validation, review, and refinement of content; and aided in dissemination. All partners contributed to coordination and cohesion of content, supporting alignment of messages, and helping to minimize redundancy. In conjunction with senior leaders at the CEP’s organization, oversight was provided by a Clinical Working Group (CWG) composed of 10 primary care providers and key stakeholders. An Advisory Committee consisting of clinicians, system and partner representatives and a patient advisor from across Ontario in both rural and urban communities provided expert guidance, insight and validation for content. The MOH, CWG and Advisory Committee contributed to the development of the CRC and assisted with dissemination.

Close collaborating partners throughout the life of the CRC included the McMaster University Department of Family Medicine, the Ontario College of Family Physicians (OCFP), the Nurse Practitioner’s Association of Ontario (NPAO), the Ontario Medical Association (OMA), and the Section for General & Family Practice (SGFP). Additional expert partners and collaborators included (but were not limited to), family physicians and nurse practitioners, the Provincial Primary Care Advisory Table (PPCAT), the Ontario Primary Care Council (PCC), the Office of the Chief Medical Officer of Health (CMOH), health system leaders, faculty from leading Canadian medical schools, staff in relevant provincial ministries and bodies, patient experts, and leadership and staff of professional organizations serving our target audience.

## Methods

**Fig. 7:**
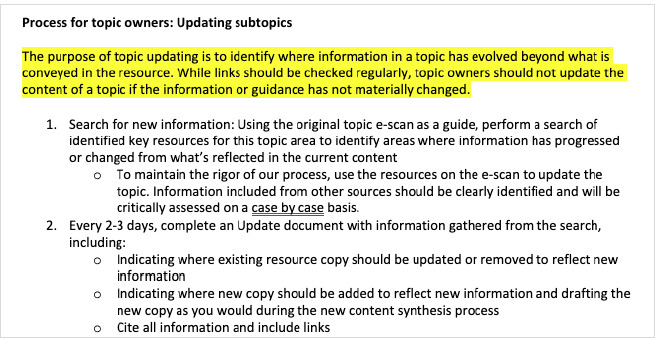
Update process excerpt

**Fig. 8:**
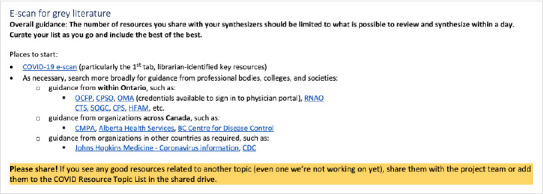
Rapid Resource Development Protocol excerpt

**Fig. 9:**
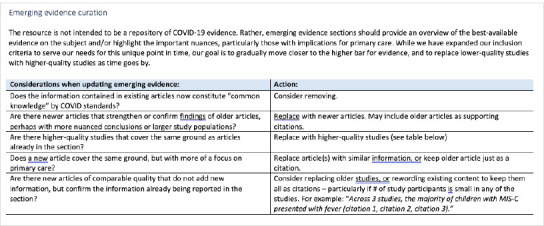
RRDP-evidence curation excerpt

CEP’s standard clinical tool development methodology prioritizes confidence in conclusions and low risk of bias in evidence. Typically, systematic reviews are preferred to individual studies, and specific critical appraisal instruments are used to inform inclusion and exclusion of systematic reviews and Clinical Practice Guidelines (CPGs). But due to the rapidly evolving nature of COVID-19 information, our standard criteria were insufficient to meet the demands of the pandemic. In developing the CRC, the task for the Director of Information Services was to design a methodology that would generate the most authoritative evidence syntheses conceivable within an accelerated content development timeframe of one to three days. Three tools were created to inform the inclusion, evaluation, curation, and synthesis of COVID-19 information (Figures 7-9):

1. A Rapid Resource Development Protocol Adapted from the U.K.’s National Institute for Health and Care Excellence (NICE)’s 2020 “Interim process and methods for guidelines developed in response to health and social care emergencies,” the protocol emphasized the use of existing guidance to avoid duplication and promote consistency while ensuring rapid dissemination of critical information to healthcare providers. It included style guidelines and a new citation format [2].
2. Inclusion/Exclusion Criteria: Peer-reviewed individual studies were included if their incorporation would offer valuable insights without misleading primary care providers. Non-peer-reviewed studies and preprints were excluded. In relevant CRC sections, a disclaimer was posted instructing clinicians on how to interpret included studies:
  **There is only low-quality evidence available on COVID-19, as it is an emerging virus**. Many studies being released have not been peer-reviewed. Among those that have been peer-reviewed, many are small, retrospective observational studies and thus have serious limitations and risks of bias. **While the findings of emerging COVID-19 studies can be useful in helping to broaden our understanding about how the virus might operate, the results of COVID-19 studies should not be considered validated**.
3. A Resource Update Process was designed to keep the CRC reflective of the latest shifts in guidance for primary care providers by updating and replacing linked resources as newer or better ones became available.

The methodology represented a dynamic and responsive approach to managing and disseminating healthcare information during the pandemic, an integrated strategy involving careful selection of evidence, regular updates to reflect new insights, and adherence to quality standards to support healthcare providers in delivering care.

### Content development

For each topic and subtopic, an owner and coordinator were responsible for tracking the topic and identifying updates. This included an often-daily search for new information using key resources identified during the initial topic environmental scan, a review or refresh of the content based on new findings, and then a critical assessment of these updates by Information Services team members before submission for publication on the CRC.

Growing and maintaining the CRC was resource-intensive, with an internal staff complement at times reaching 10-20 full-time equivalents (FTEs). Numerous project management tools were developed to track content development for the CRC, including a master list of topics and subtopics, owners and coordinators, and date of last update. Using conditional formatting, topics would show up in green if it was <2 days since an update; yellow if it was 2-3 days since an update; and red if it was >3 days since an update.

## Outcomes

**Fig. 10:**
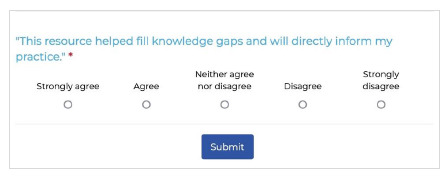
Example of pop-up survey on CRC

During its four-year operation, the CRC garnered significant attention, with close to 185,000 individuals visiting the site in total and averaging approximately 4,500 visitors per month. This level of site traffic underscores the CRC's substantial usage and value throughout the pandemic. Notably, the engagement with the CRC marked a significant increase in CEP’s website visitor retention compared to pre-pandemic levels; whereas the CEP’s other online tools typically experienced a return rate of one in five viewers, the launch of the CRC saw this figure rise to one in three within just a month. An embedded pop-up survey on the CRC's website revealed that 89% of clinician visitors felt the resource enhanced their understanding of COVID-19-specific evidence and policies (Figure 10). Additionally, the vaccine information provided on the CRC was reported to directly influence the clinical practices of 87% of the clinicians who accessed it. These metrics not only demonstrate the CRC's pivotal role in bridging knowledge gaps but also reflect the high level of satisfaction among its audience, affirming its effectiveness in supporting patient care during the pandemic.

Examples of the adoption and promotion of resources, as well as requests for collaboration, serve as qualitative illustrations of the CRC’s engagement, reach, and credibility among healthcare professionals.

Engagement: Early in its deployment, the CRC integrated a feature enabling clinicians to directly submit questions and content suggestions to the team. Within the initial eight weeks alone, more than 40 queries were addressed by the CRC team and directly influenced the resource's content evolution.

**Fig. 11:**
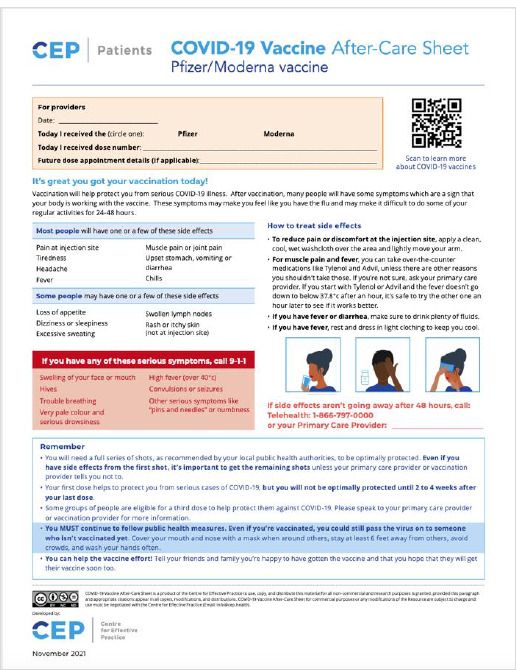
Pfizer/Moderna Vaccination Aftercare Sheets

Adoption: The CRC’s print-ready patient-focused vaccine aftercare sheets were widely adopted in prominent healthcare settings throughout the Greater Toronto Area, including St. Joseph's Hospital, Woodbine Racetrack pop-up clinic, University Health Network – MaRS Building, and Toronto General Hospital (Figure 11). The Woodbine Racetrack clinic alone administered 15,300 doses of the Pfizer vaccine in a six-day clinic [3].

Promotion: The precision and utility of the “Vaccines-at-a-Glance” two-page PDF prompted a group of family physicians of Indian origin to request support in creating analogous vaccine resources for a virtual care program in India, demonstrating the CRC's significant impact and the high level of trust it commanded in the healthcare community.

Collaboration: By summer 2020, collaboration and partnership expanded to include key organizations in primary care, including the Section on General and Family Practice (SGFP), the Ontario Medicine Association (OMA), and the University of Toronto’s Department of Family and Community Medicine (DFCM). Additionally, the CEP was creating operational guidance commissioned by the MOH’s newly formed Provincial Primary Care Advisory Table (PPCAT).

## Discussion

The CRC represented the CEP’s inaugural venture into real-time, rapid-response, evidence-based tool creation. The distinct approach of quick turnaround, rapid and candid synthesis, creative innovation, and meaningful collaboration with system partners created a unique product at a critical time. As a result, the CRC strengthened our credibility with our audience and in the broader health system, carved a path for new products, and fostered important new partnerships.

### Lessons learned

At its height, the CRC was incredibly resource intensive. Chief among the challenges of real-time public health rapid response is an unpredictable information landscape. When critical developments occurred, staff assigned to the CRC had to pivot from other work and priorities, often with no warning. While this was a challenging aspect of CRC work, our commitment to update the CRC within 24 hours of any major update to guidance led to a deep level of trust and satisfaction with our audience. In plans for future non-COVID-19 rapid resources, careful capacity planning to allow room to absorb sudden changes is a priority for the CEP.

**Fig. 12:**
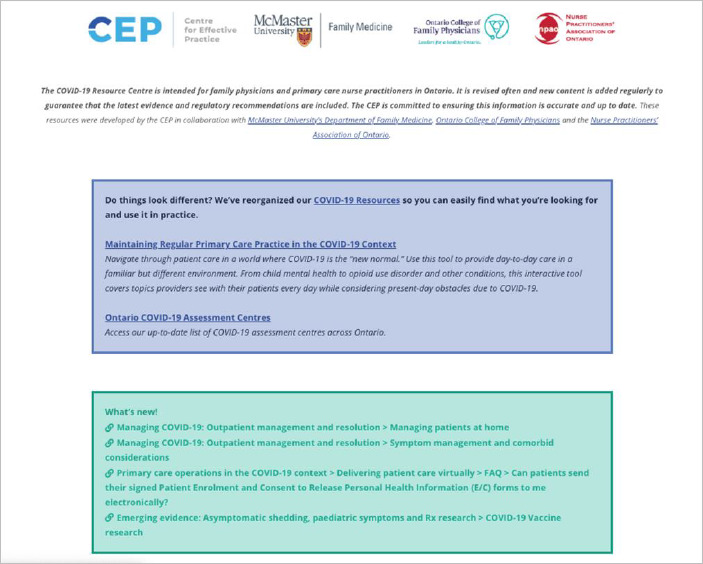
Example of highlighted boxes that indicate updated content at the top of resource

While the HTML platform was adequate for the needs of the CRC at the time, feedback received from clinicians pointed to the need for enhanced search functionality and a more powerful visual indicator to show updates and changes. To meet our audience’s needs during the pandemic, we developed a highlighted section at the top of each resource page that outlined updated content (Figure 12). In the future, we would be keenly interested to explore platforms that allowed in-text highlighting or other features that would clearly delineate new content.

### Reflections on the value of librarianship

**Fig. 13:**
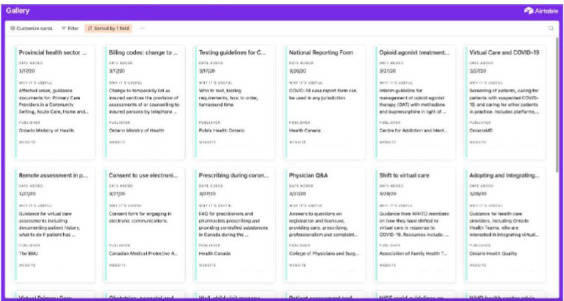
Early iteration of the CRC – 30 March 2020

While this article concentrates on the final iteration of the CRC, examining the initial phases of development—marked by trial and error—reveals significant opportunities for enhancing service delivery through critical appraisal, evidence synthesis, and knowledge translation. The first iteration of the CRC was an interactive map identifying COVID-19 assessment centers across Ontario, introduced on March 16, 2020, one day before a state of emergency was declared in Ontario. At that time, there was no comprehensive, coordinated guide available, and our primary competitor was a simple Google map. Recognizing the imperative to elevate and disseminate information from reliable sources, we supplemented the map with a page of meticulously curated, high-quality evidence. This page succinctly communicated essential information, including a brief rationale for each resource's utility to primary care clinicians (Figure 13.) However, as the volume of information expanded, the collection became unwieldy and less practical for clinicians pressed for time and unable to sift through extensive documentation or deduce practice implications.

Our response to these challenges was informed by a profound understanding of our audience's needs and our professional capacity for appraising and synthesizing evidence. By undertaking comprehensive analysis on behalf of clinicians, we enabled them to concentrate on patient care. Our adaptability, coupled with a steadfast dedication to methodological rigour, positioned us as a trusted resource among our audience. The experience of developing the CRC over four years has been immensely instructive for the Information Services team. It has not only enriched our expertise in dealing with emerging and scant evidence but also deepened our comprehension of how best to support primary care in the face of health crises. This journey underscores the enduring relevance and potential of librarianship to adapt, innovate, and lead in the ever-evolving landscape of information services and healthcare support.
